# Quantification of Extramyocellular Lipids and Intramuscular Fat from Muscle Echo Intensity in Lower Limb Muscles: A Comparison of Four Ultrasound Devices against Magnetic Resonance Spectroscopy

**DOI:** 10.3390/s23115282

**Published:** 2023-06-02

**Authors:** Enzo Piponnier, Masaki Ishikawa, Yoko Kunimasa, Kanae Sano, Kévin Jagot, Nathalie Boisseau, Toshiyuki Kurihara, Vincent Martin

**Affiliations:** 1 Université Clermont Auvergne, AME2P, F-63000 Clermont-Ferrand, France; 2 Laboratory of Human Motricity, Heath and Sport Expertise (LAMHESS), UPR 6312, University Côte d’Azur, 06108 Nice, France; 3 Graduate School of Sport and Exercise Sciences, Osaka University of Health and Sport Sciences, Osaka 590-0496, Japan; 4 Faculty of Education, Niigata University, Niigata 950-2181, Japan; 5 Faculty of Health Sciences, Morinomiya University of Medical Sciences, Osaka 559-8611, Japan; 6 Faculty of Sport and Health Science, Ritsumeikan University, Kusatsu 525-8577, Japan; 7 Faculty of Science and Engineering, Kokushikan University, Tokyo 154-8515, Japan; 8 Institut Universitaire de France (IUF), F-75005 Paris, France

**Keywords:** intramuscular fat, muscle quality, magnetic resonance imaging

## Abstract

This study aimed to compare different ultrasound devices with magnetic resonance spectroscopy (MRS) to quantify muscle lipid content from echo intensity (EI). Four different ultrasound devices were used to measure muscle EI and subcutaneous fat thickness in four lower-limb muscles. Intramuscular fat (IMF), intramyocellular (IMCL) and extramyocellular lipids (EMCL) were measured using MRS. Linear regression was used to compare raw and subcutaneous fat thickness-corrected EI values to IMCL, EMCL and IMF. IMCL had a poor correlation with muscle EI (r = 0.17–0.32, NS), while EMCL (r = 0.41–0.84, *p* < 0.05–*p* < 0.001) and IMF (r = 0.49–0.84, *p* < 0.01–*p* < 0.001) had moderate to strong correlation with raw EI. All relationships were improved when considering the effect of subcutaneous fat thickness on muscle EI measurements. The slopes of the relationships were similar across devices, but there were some differences in the y-intercepts when raw EI values were used. These differences disappeared when subcutaneous fat thickness-corrected EI values were considered, allowing for the creation of generic prediction equations (r = 0.41–0.68, *p* < 0.001). These equations can be used to quantify IMF and EMCL within lower limb muscles from corrected-EI values in non-obese subjects, regardless of the ultrasound device used.

## 1. Introduction

The prevalence of obesity and overweight is increasing worldwide. According to the World Health Organization, 39% of adults aged 18 or over were overweight and 13% were obese in 2016. This population is affected by numerous complications and comorbidities [1,2,3,4,5,6] that may result from the accumulation of fat within and around tissues. Accumulation of abdominal fat and particularly visceral fat in obese people [7,8], is associated with metabolic syndrome and cardiovascular risks [2,6,8,9,10]. Ectopic adipose tissue can also be located within the muscle in obese people [11,12] but also in myopathic patients [13,14], elderly [15,16] or after a period of inactivity [17] and represents a risk for metabolic disorders [11,18] and muscle function [19]. As such, it is generally considered that fat deposit generates lipotoxicity [20].

It is essential to be able to assess intramuscular fat (IMF) content in clinical practice. This fat depot is composed of fat located either within the muscle fibers (intramyocellular lipids; IMCL) or between the muscle fibers (extramyocellular lipids; EMCL). The latter can also be found between and around skeletal muscles and constitutes a genuine adipose tissue referred to as intermuscular fat adipose tissue (IMAT) [19,21] when evaluated with magnetic resonance imaging (MRI-T1-sequence or mDixon technique) or computed tomography. The location of the lipid depot has clinical significance: IMCL content is known to be positively correlated with insulin resistance [22,23] and negatively correlated to muscle function [24]. EMCL and IMAT have also been associated with dysglycaemia, including insulin sensitivity and glucose tolerance [21], and with reduced muscle function [25], but their effects seem distinct from those of IMCL [21]. In addition, the athlete paradox revealed that chronic exercise training actually increased the muscle triglyceride component of IMCL, but this effect was independent of exercise effects on IMAT/EMCL [26]. In clinical practice, it is therefore important to distinguish the accumulation of IMCL and EMCL.

Proton magnetic resonance (^1^H-MRS) stands out as the more accurate and convenient non-invasive method to quantify IMCL and EMCL [27]. Unfortunately, this is an expensive method that limits its use in daily clinical practice. Qualitative grayscale analysis, known as muscle echo intensity (EI) appears the less expensive and the most accessible alternative [27]. Some studies showed that increased EI is negatively correlated to muscle strength [28,29,30]. Initially, EI was used in agribusiness to identify meat quality and the percentage of IMF of pig and beef meat [31,32]. However, EI is only a qualitative approach. To overcome this limitation and provide a quantitative evaluation of IMF, one study related IMF content assessed by MRI to EI measured by an ultrasound device in brightness mode (B-mode) [33]. Prediction equations were generated between muscle EI and IMF. As subcutaneous fat thickness can alter or reflect the absorption of ultrasound waves in deeper tissues [34,35], the authors took this parameter into account in their equations, which significantly improved the prediction. However, this study did not differentiate IMCL and EMCL in the quantification of IMF. To date, only one study attempted to quantify IMCL and EMCL from EI in the vastus lateralis (VL) and the biceps femoris (BF). Akima et al. reported significant correlations between EI and EMCL but did not observe significant relationships between EI and IMCL. This could be accounted for by the low spatial resolution of ultrasound imaging or the fact that these authors did not account for the potential confounding effect of subcutaneous fat thickness [36]. Another limitation of these studies is that they propose prediction equations that are specific to a particular ultrasound device. As proposed by Young et al. [33], it would be advisable to test other devices because the muscle EI values depend on ultrasound transducer characteristics and image post-processing techniques. Pillen et al. [34] demonstrated that although ultrasound devices create images that are different on a pixel level, EI can be comparable between ultrasound devices on a macro level, after the application of a conversion factor. That would allow the creation of generic prediction equations relating IMCL, EMCL and IMF to EI. 

The purpose of the present study was to compare four ultrasound devices against MRI to quantify IMCL, EMCL and IMF of two plantar flexors [gastrocnemius medialis (GM) and soleus (SOL)] and two knee extensor [VL and vastus medialis (VM)] muscles from EI, corrected for subcutaneous fat thickness and to generate prediction equations.

## 2. Materials and Methods

### 2.1. Subjects

A total of thirty subjects (11 women and 19 men) were recruited in the present study. Participants displayed several body mass index (BMI) values and disparate physical activity levels (from sedentary to highly endurance-trained participants), in order to obtain a varied sample of muscle adiposity. The study was conducted in accordance with the Declaration of Helsinki and approved by the ethics committee of Ritsumeikan University (BKC-IRB-2018-062). All the participants were fully informed and gave their written consent before any testing was conducted.

### 2.2. Design

Participants were asked to come to the laboratory on one occasion, to undergo all the testing procedures. The ultrasound tests were performed first and then the MRS measurements. This allowed us to perform the MRS scanning in the specific muscle area assessed by ultrasound. Body composition was also assessed during this experimental session.

### 2.3. Body Composition Assessment

Whole-body and right leg fat-free mass and fat mass were assessed by impedancemetry (InBody 770, InBody co., Seoul, Republic of Korea).

### 2.4. Ultrasound Experimental Measurement and Analysis

**Figure 1:**
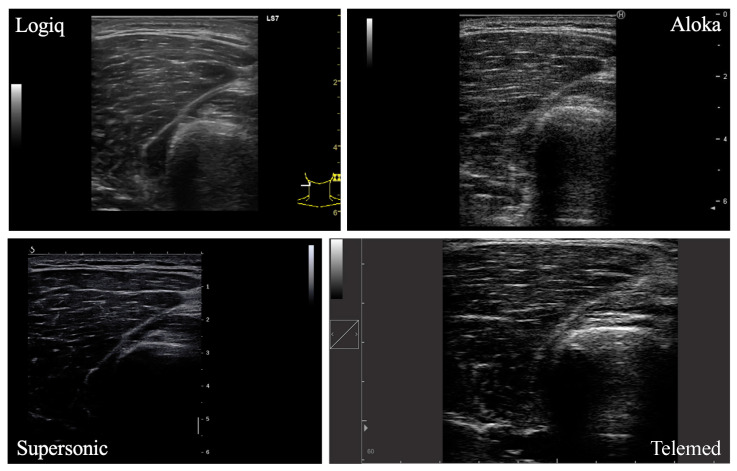
Ultrasound images of the vastus lateralis muscle from four different devices.

A total of four lower limb muscles were investigated in this study (VM, VL, GM, SOL; Figure 1). In each participant, the right leg was assessed by the same experimenter in a resting, supine position on an examination table. Four ultrasound devices, differing in terms of post-processing techniques and transducer characteristics, were used for analysis: LOGIQ (Logiq S7, GE Health Care, Chicago, IL, USA), SUPERSONIC (Aixplorer, Supersonic Imagine, Aix-en-Provence, France), ALOKA (Noblus, Hitachi–Aloka Medical Japan, Tokyo, Japan) and TELEMED (Echo Blaster 128 CEXT-1Z, Telemed Ltd., Vilnius, Lithuania) devices. Real-time brightness mode (B-mode) was used for all devices with specific parameters. Settings for each ultrasound device are reported in Table 1. The scanning depth was adjusted for participants with greater subcutaneous fat thickness to gather maximal muscle area. The other parameters were set in order to obtain a clear visualization of the muscle under consideration and its aponeuroses.

To normalize measurements, the leg and thigh lengths were measured. Thigh length was defined as the distance between the antero-superior iliac spine and the superior lateral aspect of the patella. Leg length was defined as the distance between the inferior lateral aspect of the patella and the calcaneus. Ultrasound scans were performed between the lower- and middle-third of the thigh (for VM and VL, respectively) and between the third and fourth quarter of the leg length (for GM and SOL, respectively). These anatomical landmarks were marked on the skin with permanent ink during ultrasound scanning and later with oil capsules taped to the skin during MRS scanning. Ultrasound scanning was performed with a generous amount of ultrasound gel applied to the probe to avoid pressure on the skin and improve imaging quality. Each muscle was scanned three times for each ultrasound device and each participant. A total of forty-eight scans were performed for each participant. Images were exported to a jpeg format.

ImageJ (U.S. National Institutes of Health, Bethesda, MA, USA) was used for image analysis. Muscle EI was assessed using grayscale calculation by the same experimenter. For each image, three values of muscle EI, examined muscle area and subcutaneous fat thickness were calculated. For each muscle and ultrasound device, the average of nine image values (from muscle EI, area and subcutaneous fat thickness) was computed for further analysis.

### 2.5. MRS Acquisition and Analysis

**Figure 2:**
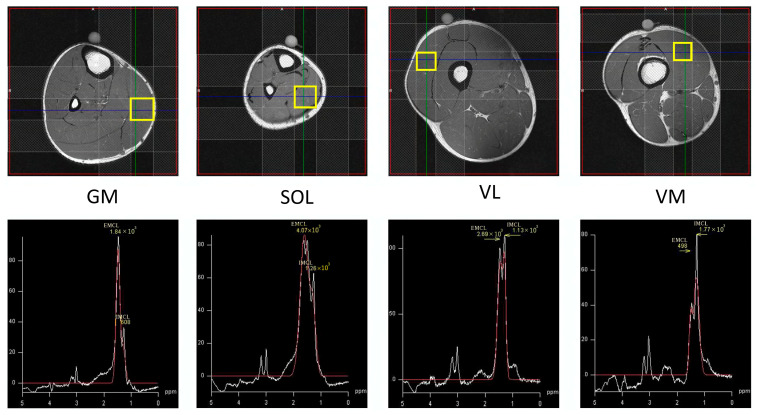
Upper row: cross-sectional imaging of the gastrocnemius medialis (GM), soleus (SOL), vastus lateralis (VL) and vastus medialis (VM) muscles; the yellow squares indicate the voxels evaluated with proton magnetic resonance spectroscopy lower row: typical examples of ^1^H-MR spectra for the gastrocnemius medialis (GM), soleus (SOL), vastus lateralis (VL) and vastus medialis (VM) muscles.

Non-invasive ^1^H-MRS measurement of the muscles (VM, VL, GM, SOL; Figure 1) was performed on a 1.5T MR system (Signa HDxt, GE Medical Systems, Buckinghamshire, England) with an 8ch body array coil. The subject lay in the supine position with their right leg carefully positioned parallel to the main magnetic field. Single voxel MRS measurement was performed using the Point Resolved Spectroscopy (PRESS) sequence (TR/TE 2000/35 ms, 20 × 20 × 20 mm^3^, 32 acquisitions). All subjects were instructed to maintain their usual physical activities and dietary habits for a few days before the measurement. Outer volume suppression slabs were used around the voxel of interest to diminish the signal of subcutaneous fat located nearby the voxel. The analyses and estimation of the absolute concentration of IMCL and EMCL were accomplished by LCModel software ver6.3 with Weis customized calculation [37]. An unsuppressed water signal was used as an internal standard. During data processing T1 and T2 relaxation effects of the water reference were taken into account with the LCModel’s control parameter, atth2o, determined using the following equation: exp (−TE/T2) [1−exp (−TR/T1)] [37,38]. The peak chemical shift of IMCL was adjusted as 1.3 ppm and that of EMCL was 1.5 ppm. Concentrations of EMCL and IMCL were computed as millimoles per liter of muscle tissue (mM) and converted into millimoles per kg wet weight as recommended by Weis et al. [37]. Finally, IMF was calculated as the sum of EMCL and IMCL. Typical example spectra are illustrated in Figure 2.

### 2.6. Muscle and Echographer-Specific Equations

Correlations between raw EI and IMCL, EMCL and IMF from MRS were made for all the muscles for each ultrasound device. To account for the potential confounding effect of subcutaneous fat thickness on muscle EI, the correction factor proposed by Young et al. [33] was also applied to raw EI to generate new equations, corrected for subcutaneous fat thickness, as proposed in the equation below.

y_2_ = y_1_ + (x × cf) (1)

where y_2_ is the corrected EI, y_1_ is the raw EI, x is the subcutaneous fat thickness and cf is the Young correction factor [33], i.e., 40.5278.

### 2.7. Statistics

Linear regression models were used to build, examine and compare relationships between muscle EI and fat content (IMF, IMCL, EMCL) for all muscles and ultrasound devices. The strength of the positive and negative correlations were evaluated using the Evans [39] guidelines: very weak: 0.00–0.019, weak: 0.20–0.39, moderate: 0.40–0.59, strong: 0.60–0.79, very strong: 0.80–1.0. Then, for significative relationships, ANOVAs were conducted to compare the slopes and Y-intercepts between relationships. When the ANOVA revealed a significant difference, a Tukey test was used as a post-hoc test.

Examined muscle area, raw and corrected EI and fat thickness were also compared between devices. Distribution normality and homogeneity of variances of these variables were tested using a Shapiro–Wilk normality test and Bartlett’s test, respectively. Then, one-way ANOVAs with repeated measures were run. When the ANOVA revealed a significant difference, a Tukey test was used as a post-hoc test. Statistical analyses were conducted on GraphPad 7 (GraphPad Software, San Diego, CA, USA). Values are expressed as mean ± standard deviation (SD). The α-level of significance was set at *p* < 0.05.

## 3. Results

### 3.1. Subject’s Characteristics

Physical characteristics of the participants are described in Table 2.

### 3.2. MRS and Ultrasound Parameters

The IMCL, EMCL and IMF values for GM, SOL, VL and VM from MRS are represented in Table 3.

The examined muscle area, raw EI, Young corrected EI and subcutaneous fat-thickness for GM, SOL, VL and VM from the four ultrasound devices, are shown in Table 4. As expected, raw and corrected EI differed between ultrasound devices for the four investigated muscles.

### 3.3. Ultrasound Devices vs. MRS Linear Regressions

Significant moderate to very strong correlations were found between raw EI and IMF and between raw EI and EMCL for the four muscles in Logic, Aloka and Telemed ultrasound devices. In addition, only significant moderate and strong correlations were found for GM and VL, respectively, with the Supersonic ultrasound device (Table 5). No significant correlation was found between raw EI and IMCL whatever the muscles or ultrasound devices considered.

Significant moderate to very strong correlations were found between Young corrected EI and IMF and between Young corrected EI and EMCL for the four muscles with the four ultrasound devices, except between Young corrected EI and EMCL for SOL in Supersonic device (Table 6). Significant moderate correlations were found between Young corrected EI and IMCL only for VM in Logic, Aloka and Supersonic ultrasound devices.

**Figure 3:**
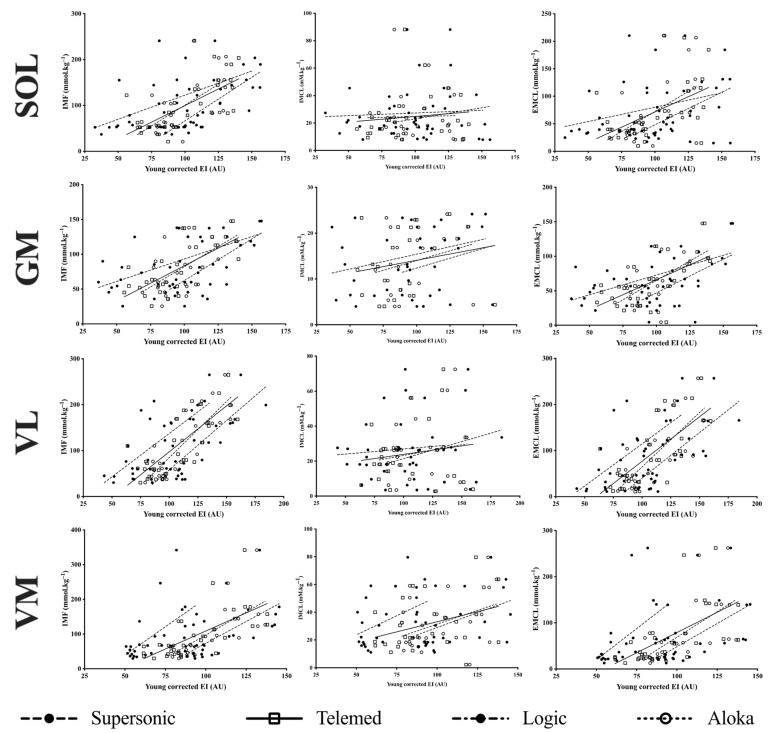
Correlations between intramuscular fat (IMF; left panels) and extramyocellular lipids (EMCL; right panels) measured by MRS and raw echo intensity (EI—1st and 3rd column) and Young corrected echo intensity (EI—2nd and 4th column) measured with ultrasound devices in the soleus (SOL), gastrocnemius medialis (GM), vastus lateralis (VL) and vastus medialis (VM) muscles.

For example, Figure 3 shows raw EI/IMF, raw EI/EMCL, Young corrected EI/IMF and Young corrected EI/IMF correlations for VL in the four ultrasound devices.

### 3.4. Comparisons of the Calibration Equations between Ultrasound Devices

Anecdotal differences between some devices were found in slopes and Y-intercepts for raw EI/IMF and raw EI/EMCL relationships in GM, SOL and VL (Supplementary Data S1–S4). However, no difference between devices was observed when Young corrected EI was computed against MRS data (Supplementary Data S1–S4).

Consequently, the data from the four devices were averaged for each participant and generic equations were established for each muscle from these average values (Table 7).

## 4. Discussion

The purpose of the present study was to compare prediction equations of IMCL, EMCL and IMF from EI between four ultrasound devices. IMCL was poorly correlated with muscle EI, whereas EMCL and IMF were moderate to strongly correlated with muscle EI of GM, SOL, VL and VM. All the relations were improved using the Young corrected factor, showing the potential confounding effect of subcutaneous fat thickness on muscle EI measurements for all muscles. Slopes of the relationships were comparable between the four devices, but y-intercepts differed, with few exceptions. Interestingly, these differences disappeared when Young corrected EI were computed against MRS data, giving the possibility to generate generic prediction equations.

### 4.1. Quantification of IMF, IMCL and EMCL with Ultrasound

As far as we know, only one study tried to quantify EMCL and IMCL using muscle raw EI [36]. The authors found a moderate correlation between raw EI and EMCL in VL and BF (r = 0.65). In the present study, r values were greater for all the ultrasound devices concerning VL but comparable for other muscles investigated such as GM (excepted for Supersonic), SOL (excepted for Supersonic) and VM. Results from this experiment confirm that raw EI values can be used to quantify EMCL.

Conversely, Akima et al. [36] failed to find correlations between IMCL and raw EI in VL and BF muscles. The lack of correlation could be explained by the low spatial resolution of ultrasound imaging or by the fact that these authors did not account for the potential confounding effect of subcutaneous fat thickness [36]. Indeed, Young et al. [33] found an independent influence of subcutaneous fat thickness on muscle EI, with alteration of reflection or absorption of the ultrasound waves in deeper tissues [34,35]. Consequently, Young et al. took the subcutaneous fat thickness into account in their prediction equations. This improved the quantification of IMF from EI values. In the current study, we applied this correction factor for the prediction of EMCL and IMCL. This translated into a modest increase in the strength of the relationship between EMCL and muscle EI but failed to improve the relationship between corrected muscle EI and IMCL. It can be concluded that the lack of correlation between EI and IMCL seems to be explained by the low spatial resolution of ultrasound imaging rather than the confounding effect of subcutaneous fat thickness.

Nevertheless, one should not conclude about the complete inability of ultrasound imaging to quantify IMCL. The EI-IMF relationships were stronger than EMCL-EI relationships, suggesting that ultrasound imaging can quantify at least part of the IMCL included in the IMF content. This good ability of ultrasound imaging to quantify IMF is consistent with previous studies comparing muscle EI to the percentage of IMF quantified from a muscle biopsy sample [40,41] or from MRS data [36]. As previously mentioned, corrected EI from subcutaneous fat thickness improved EI-IMF relationship quality. Indeed, our results showed moderate to very strong correlations for Young corrected EI and IMF relationship in GM, SOL, VL and VM.

### 4.2. Comparison between US Devices

The ability to quantify muscle fat content from EI may be ultrasound device dependent. The present study is the first to compare different ultrasound devices to assess EMCL and IMF in lower limb muscles. Some anecdotal differences were observed in y-intercept and slopes between devices when computing raw EI values to derive raw EI-IMF and raw EI/EMCL relationships in GM, SOL and VL. This may be ascribed to the fluctuations of ultrasound waves encountering different tissues [14] and the subsequent image processing, which is specific to each device. Interestingly, the EI-EMCL and EI-IMF relationships did not differ between ultrasound devices when the subcutaneous thickness was considered. This allowed the production of generic equations for the indirect quantification of IMF and EMCL from Young corrected EI data. This result has important practical implications. Indeed, several studies have assessed muscle quality from EI and their relation with muscle strength and function in several populations [14,28,29,42]. However, this qualitative approach is limited by the fact that EI values are not comparable between ultrasound devices and thus between studies unless a conversion factor is given, which is currently unavailable in the literature. That would require correcting EI values obtained from several devices and settings [34,43]. Interestingly, the current results suggest that correcting EI values for the confounding influence of subcutaneous fat thickness allows a generic and quantitative approach to muscle fat content. This opens new avenues for clinicians and scientists interested in the impact of muscle fat content on physical function [44].

### 4.3. Limitations

Some limitations of our experimental approach should be acknowledged. First, we investigated two plantar flexors and two knee extensor muscles. The proposed equations are muscle-specific and need to be extended to other muscles, in particular the dorsi-flexors and knee flexors and upper limb muscles which are seldom studied. Second, unlike Young sex-specific equations [33], our participants’ sampling did not allow us to generate sex-specific equations. Third, the muscle area examined for EI measurement was bigger than the muscle area examined with MRS, because it was impossible to reproduce exactly the location of the MRS examination zone with ultrasound, although attention was paid to evaluating the same location (slice) in the longitudinal direction. For EI measurements, we chose to examine a muscle area that would encompass the MRS area. Given the heterogeneity of EMCL/IMF distribution within muscles, the correlation coefficients between EI and EMCL/IMF measurements would have been stronger, should the EI measurements be performed on the same voxels used for MRS. Fourth, the purpose of this study was not to uniformize the settings and images between US systems, but to compare four ultrasound devices against MRI, implying that settings would differ between systems. This study was thus designed in an ecological way. The settings were chosen for each system with the aim of producing the clearest possible image (as practiced in the clinical context). However, to improve the approach of the current study, normalization to a reference material (*i.e.,* calibration phantom) is required. That would reduce system-dependent variance, render the data fully independent of instrumental settings and ultimately would allow producing universal equations for the quantification of muscle fat content. Finally, it remains unknown if the generic equations produced in the current study are valid for populations such as severely obese patients, where a very high subcutaneous fat thickness may potentially impair the ability to quantify IMF and EMCL from EI values.

## 5. Conclusions

From a practical point of view, it can be concluded that ultrasound imaging can be used to indirectly quantify EMCL and IMF from EI in non-obese people, independently of the ultrasound device type, provided that the subcutaneous fat thickness is considered. This technique is less able to detect IMCL, owing to its low spatial resolution. IMCL can only be partly reflected in the IMF quantification from EI. However, if one wants to specifically focus on IMCL, to relate this variable to physiological aspects such as insulin sensitivity [22,23], alternative techniques such as MRS may prove more precise.

## Figures and Tables

**Table 1:** Ultrasound devices settings.

	Logiq	Aloka	Supersonic	Telemed
Probe	L3-12-D	L55	SL15-4	HL9.0/60/128Z-2
Gain	58 a.u.	10 dB	60 dB	100%
Transducer frequency	8 MHz	5 MHz	12 MHz	8 MHz
Dynamic range	63 dB	55 dB	62 dB	62 dB
Focus	50 mm	62 mm	52 mm	52 mm
Power	100%	100%	100%	77%

**Table 2:** Physical characteristics of the participants.

**Age (years)**	23.1 ± 7.1 (19–48)
**Height (cm)**	169.0 ± 6.9 (148–179.4)
**Body mass (kg)**	66.3 ± 10.8 (45.4–92.4)
**BMI (kg/m** **^2^)**	23.2 ± 2.9 (19.4–29.7)
**Fat mass (%)**	20.6 ± 8.5 (8.2–34.5)
**Fat mass in the right leg (%)**	20.8 ± 8.0 (9.7–35.2)

BMI: Body Mass Index. Values are expressed as mean ± SD (minimum value-maximum value).

**Table 3:** MRS imaging parameters.

	GM	SOL	VL	VM
IMF (mmol.kg^−1^)	1.70 ± 0.76	2.27 ± 1.27	2.57 ± 1.64	2.26 ± 1.62
IMCL (mmol.kg^−1^)	0.34 ± 0.17	0.59 ± 0.44	0.59 ± 0.44	0.74 ± 0.45
EMCL (mmol.kg^−1^)	1.36 ± 0.68	1.68 ± 1.20	1.98 ± 1.67	1.52 ± 1.39

IMF: intramuscular fat; IMCL: intramyocellular lipids; EMCL: extramyocellular lipids; GM: gastrocnemius medialis; SOL: soleus; VL: vastus lateralis; VM: vastus medialis; Values are expressed as mean ± SD.

**Table 4:** Ultrasound devices parameters.

		Logic	Aloka	Supersonic	Telemed
GM	Examined muscle area (cm^2^)	7.2 ± 1.3	7.0 ± 1.0	7.3 ± 1.1	7.5 ± 1.3
	Raw EI (AU)	89.5 ± 11.7	71.8 ± 12.4 *	56.4 ± 25.0 *^,#^	69.1 ± 18.6 *^,†^
	Young corrected EI (AU)	117.6 ± 20.9	98.4 ± 20.7 *	82.1 ± 32.6 *^,#^	97.3 ± 27.5 *^,†^
	Fat thickness (cm)	0.7 ± 0.3	0.7 ± 0.2	0.6 ± 0.2	0.7 ± 0.3
SOL	Examined muscle area (cm^2^)	6.1 ± 1.3	5.7 ± 1.2	5.6 ± 1.3	5.9 ± 1.7
	Raw EI (AU)	82.2 ± 12.9	62.0 ± 14.7 *	45.1 ± 25.0 *^,#^	64.4 ± 17.8 *^,†^
	Young corrected EI (AU)	117.7 ± 21.4	96.6 ± 21.2 *	79.3 ± 29.8 *^,#^	98.5 ± 25.9 *^,†^
	Fat thickness (cm)	0.9 ± 0.3	0.9 ± 0.2	0.8 ± 0.2	0.8 ± 0.2
VL	Examined muscle area (cm^2^)	9.3 ± 1.9	9.1 ± 1.5	9.1 ± 1.8	10.2 ± 1.7 *^,#,†^
	Raw EI (AU)	92.7 ± 13.3	79.3 ± 13.0 *	56.1 ± 17.0 *^,#^	74.5 ± 17.1 *^,†^
	Young corrected EI (AU)	123.1 ± 24.6	110.0 ± 23.6 *	84.7 ± 25.0 *^,#^	105.1 ± 27.8 *^,†^
	Fat thickness (cm)	0.8 ± 0.3	0.8 ± 0.3	0.7 ± 0.3	0.8 ± 0.3
VM	Examined muscle area (cm^2^)	11.0 ± 2.3	11.2 ± 1.9	10.3 ± 1.7 ^#^	12.6 ± 1.9 *^,#,†^
	Raw EI (AU)	73.7 ± 9.1	63.7 ± 10.5 *	33.9 ± 6.2 *^,#^	58.0 ± 14.5 *^,#,†^
	Young corrected EI (AU)	109.1 ± 19.4	98.8 ± 20.0 *	67.4 ± 14.3 *^,#^	92.9 ± 23.9 *^,#,†^
	Fat thickness (cm)	0.9 ± 0.3	0.9 ± 0.3	0.8 ± 0.3	0.9 ± 0.3

GM: gastrocnemius medialis; SOL: soleus; VL: vastus lateralis; VM: vastus medialis; EI: echo intensity; AU: arbitrary unity; *, # and †: significantly different from Logic, Aloka and Supersonic, respectively. Values are expressed as mean ± SD.

**Table 5:** Raw echo intensity calibration equations.

		Logiq		Aloka		Supersonic		Telemed	
		Calibration Equations	r	Calibration Equations	r	Calibration Equations	r	Calibration Equations	r
IMF	GM	y=0.051×x−2.550	0.58 **	y=0.056×x−2.005	0.67 ***	y=0.018×x+0.943	0.52 *	y=0.037×x−0.574	0.70 ***
	SOL	y=0.070×x−3.380	0.66 ***	y=0.056×x−1.146	0.59 ***	y=0.020×x+1.515	0.37	y=0.044×x−0.394	0.57 ***
	VL	y=0.103×x−6.906	0.84 ***	y=0.116×x−6.590	0.89 ***	y=0.057×x−0.558	0.60 **	y=0.077×x−3.194	0.80 ***
	VM	y=0.119×x−6.437	0.60 **	y=0.102×x−4.174	0.62 ***	y=0.098×x−1.006	0.34	y=0.059×x−1.119	0.49 **
IMCL	GM	y=0.003×x+0.099	0.18	y=0.003×x+0.119	0.21	y=0.002×x+0.223	0.32	y=0.002×x+0.214	0.20
	SOL	y=0.010×x−0.209	0.27	y=0.002×x+0.465	0.07	y=0.001×x+0.605	0.04	y=0.004×x+0.335	0.18
	VL	y=0.008×x−0.121	0.26	y=0.006×x+0.129	0.17	y=−0.002×x+0.731	−0.07	y=0.003×x+0.394	0.11
	VM	y=0.016×x−0.394	0.33	y=0.014×x−0.136	0.32	y=0.025×x−0.042	0.30	y=0.008×x+0.311	0.25
EMCL	GM	y=0.037×x−1.786	0.46 *	y=0.046×x−1.753	0.58 **	y=0.016×x+0.604	0.49 *	y=0.030×x−0.497	0.59 ***
	SOL	y=0.040×x−1.627	0.41 *	y=0.045×x−1.112	0.49 **	y=0.007×x+1.336	0.15	y=0.034×x−0.431	0.46 *
	VL	y=0.096×x−6.817	0.77 ***	y=0.114×x−6.971	0.84 ***	y=0.059×x−1.209	0.61 **	y=0.077×x−3.682	0.76 ***
	VM	y=0.100×x−5.772	0.56 **	y=0.088×x−3.951	0.59 ***	y=0.101×x−1.773	0.39	y=0.052×x−1.394	0.48 **

x: raw echo intensity (in AU); IMF: Intramuscular fat (in mmol.kg^−1^); IMCL: Intramyocellular lipids (in mmol.kg^−1^); EMCL: Extramyocellular lipids (in mmol.kg^−1^); GM: Gastrocnemius medialis; SOL: Soleus; VL: Vastus lateralis; VM: Vastus medialis; *, ** and *** statistically significant at *p* < 0.05, *p* < 0.01 and *p* < 0.001, respectively.

**Table 6:** Young corrected echo intensity calibration equations.

		Logiq		Aloka		Supersonic		Telemed	
		Calibration Equations	r	Calibration Equations	r	Calibration Equations	r	Calibration Equations	r
IMF	GM	y=0.029×x−1.382	0.63 **	y=0.028×x−0.811	0.60 ***	y=0.016×x+0.696	0.57 **	y=0.024×x−0.414	0.71 ***
	SOL	y=0.046×x−2.975	0.71 ***	y=0.044×x−1.930	0.67 ***	y=0.025×x+0.424	0.55 **	y=0.035×x−1.083	0.65 ***
	VL	y=0.052×x−3.787	0.79 ***	y=0.059×x−3.847	0.82 ***	y=0.046×x−1.247	0.71 ***	y=0.048×x−2.478	0.79 ***
	VM	y=0.061×x−4.302	0.66 ***	y=0.064×x−4.047	0.72 ***	y=0.079×x−2.991	0.63 ***	y=0.050×x−2.392	0.68 ***
IMCL	GM	y=0.002×x+0.091	0.24	y=0.002×x+0.132	0.24	y=0.002×x+0.215	0.30	y=0.001×x+0.207	0.21
	SOL	y=0.004×x+0.165	0.17	y=0.001×x+0.541	0.03	y=0.001×x+0.565	0.05	y=0.002×x+0.403	0.12
	VL	y=0.005×x+0.049	0.28	y=0.003×x+0.265	0.17	y=0.001×x+0.509	0.09	y=0.002×x+0.344	0.15
	VM	y=0.010×x−0.331	0.44 *	y=0.010×x−0.197	0.41 *	y=0.013×x−0.095	0.43 *	y=0.007×x+0.097	0.36
EMCL	GM	y=0.024×x−1.222	0.56 **	y=0.027×x−1.141	0.60 ***	y=0.014×x+0.369	0.55 **	y=0.021×x−0.502	0.64 ***
	SOL	y=0.029×x−1.689	0.49 *	y=0.035×x−1.767	0.57 **	y=0.013×x+0.678	0.30	y=0.027×x−0.943	0.52 **
	VL	y=0.048×x−3.799	0.71 ***	y=0.057×x−4.134	0.76 ***	y=0.044×x−1.661	0.67 ***	y=0.046×x−2.812	0.73 ***
	VM	y=0.048×x−3.679	0.57 **	y=0.053×x−3.672	0.65 ***	y=0.065×x−2.777	0.57 **	y=0.042×x−2.329	0.62 ***

x: young corrected echo intensity (in AU); IMF: Intramuscular fat (in mmol kg^−1^); IMCL: Intramyocellular lipids (in mmol.kg^−1^); EMCL: Extramyocellular lipids (in mmol.kg^−1^); GM: Gastrocnemius medialis; SOL: Soleus; VL: Vastus lateralis; VM: Vastus medialis; *, ** and *** statistically significant at *p* < 0.05, *p* < 0.01 and *p* < 0.001, respectively.

**Table 7:** Young corrected echo intensity generic calibration equations (average data from the four ultrasound devices).

	GM		SOL		VL		VM	
	Calibration Equations	r	Calibration Equations	r	Calibration Equations	r	Calibration Equations	r
IMF	y=0.026×−0.599	0.65 ***	y=0.043×x−1.904	0.68 ***	y=0.056×x−3.385	0.81 ***	y=0.063×x−3.532	0.69 ***
EMCL	y=0.023×−0.798	0.62 ***	y=0.031×x−1.387	0.52 **	y=0.055×x−3.700	0.75 ***	y=0.053×x−3.323	0.63 ***

x: young corrected echo intensity (in AU); IMF: Intramuscular fat (in (mmol.kg^−1^)); EMCL: Extramyocellular lipids (in (mmol.kg^−1^)); GM: Gastrocnemius medialis; SOL: Soleus; VL: Vastus lateralis; VM: Vastus medialis; ** and *** statistically significant at *p* < 0.01 and *p* < 0.001, respectively.

## Data Availability

All data measured and/or analyzed through the study are available from the corresponding author upon reasonable request.

## Supplementary Materials

The following supporting information can be downloaded at: https://www.mdpi.com/article/10.3390/s23115282/s1. Supplementary Data S1: Slope and Y-intercept values of the linear regressions between intramuscular fat (IMF), intramyocellular lipids (IMCL) and extramyocellular lipids (EMCL) measured by Magnetic Resonance Spectroscopy and raw echo intensity (EI) and Young corrected echo intensity (EI) measured with four ultrasound devices in the gastrocnemius medialis muscle. Supplementary Data S2: Slope and Y-intercept values of the linear regressions between intramuscular fat (IMF), intramyocellular lipids (IMCL) and extramyocellular lipids (EMCL) measured by Magnetic Resonance Spectroscopy and raw echo intensity (EI) and Young corrected echo intensity (EI) measured with four ultrasound devices in the soleus muscle. Supplementary Data S3: Slope and Y-intercept values of the linear regressions between intramuscular fat (IMF), intramyocellular lipids (IMCL) and extramyocellular lipids (EMCL) measured by Magnetic Resonance Spectroscopy and raw echo intensity (EI) and Young corrected echo intensity (EI) measured with four ultrasound devices in the vastus lateralis muscle. Supplementary Data S4: Slope and Y-intercept values of the linear regressions between intramuscular fat (IMF), intramyocellular lipids (IMCL) and extramyocellular lipids (EMCL) measured by Magnetic Resonance Spectroscopy and raw echo intensity (EI) and Young corrected echo intensity (EI) measured with four ultrasound devices in the vastus medialis muscle. Supplementary Data S5: Ultrasound images of the soleus muscle from four different devices. Supplementary Data S6: Ultrasound images of the gastrocnemius medialis muscle from four different devices. Supplementary Data S7: Ultrasound images of the vastus medialis muscle from four different devices.
