# Supplementary material for: Quantification of Extramyocellular Lipids and Intramuscular Fat from Muscle Echo Intensity in Lower Limb Muscles: A Comparison of Four Ultrasound Devices against Magnetic Resonance Spectroscopy
Source: Sensors (Basel). 2023 Jun 2;23(11):5282. doi: 10.3390/s23115282 (PMC10255973; doi:10.3390/s23115282)
Supplement: Supplementary file 1 [file sensors-23-05282-s001.zip › Supplementary_data/Supplementary_figures_captions.pdf]

Supplementary 1: Slope and Y-intercept values of the linear regressions between intramuscular fat (IMF), intramyocellular lipids (IMCL) and extramyocellular lipids (EMCL) measured by Magnetic Resonance Spectroscopy and raw echo intensity (EI) and Young corrected echo intensity (EI) measured with four ultrasound devices in the gastrocnemius medialis muscle.Significant difference: *: $P < 0.05$.

Supplementary 2: Slope and Y-intercept values of the linear regressions between intramuscular fat (IMF), intramyocellular lipids (IMCL) and extramyocellular lipids (EMCL) measured by Magnetic Resonance Spectroscopy and raw echo intensity (EI) and Young corrected echo intensity (EI) measured with four ultrasound devices in the soleus muscle.Significant difference: *: $P < 0.05$.

Supplementary 3: Slope and Y-intercept values of the linear regressions between intramuscular fat (IMF), intramyocellular lipids (IMCL) and extramyocellular lipids (EMCL) measured by Magnetic Resonance Spectroscopy and raw echo intensity (EI) and Young corrected echo intensity (EI) measured with four ultrasound devices in the vastus lateralis muscle.Significant difference: *: $P < 0.05$; **: $P < 0.01$; ***: $P < 0.001$.

Supplementary 4: Slope and Y-intercept values of the linear regressions between intramuscular fat (IMF), intramyocellular lipids (IMCL) and extramyocellular lipids (EMCL) measured by Magnetic Resonance Spectroscopy and raw echo intensity (EI) and Young corrected echo intensity (EI) measured with four ultrasound devices in the vastus medialis muscle.

Supplementary 5: Ultrasound images of the soleus muscle from four different devices.

Supplementary 6: Ultrasound images of the gastrocnemius medialis muscle from four different devices.

Supplementary 7: Ultrasound images of the vastus medialis muscle from four different devices.
