# Supplementary figures and images for: Quantification of Extramyocellular Lipids and Intramuscular Fat from Muscle Echo Intensity in Lower Limb Muscles: A Comparison of Four Ultrasound Devices against Magnetic Resonance Spectroscopy
Source: Sensors (Basel). 2023 Jun 2;23(11):5282. doi: 10.3390/s23115282 (PMC10255973; doi:10.3390/s23115282)

# RAW EI

# YOUNG EI

IMF

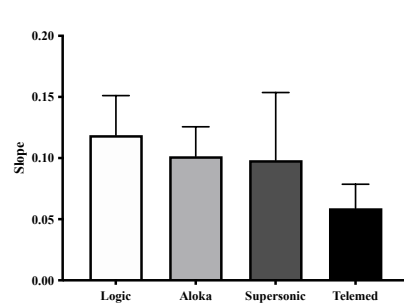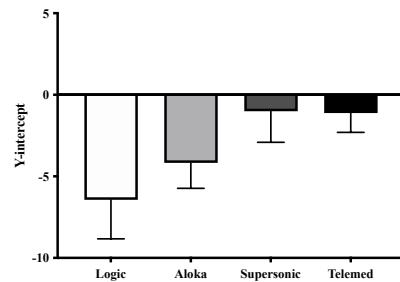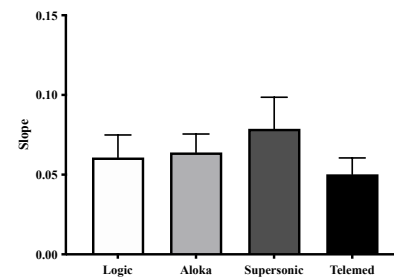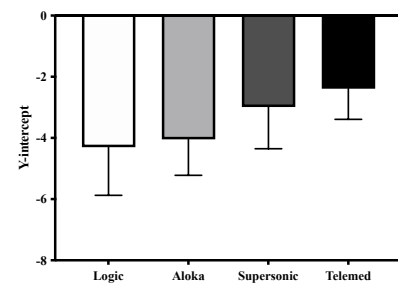

IMCL

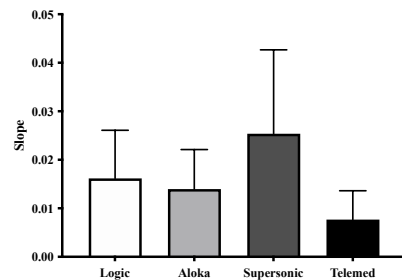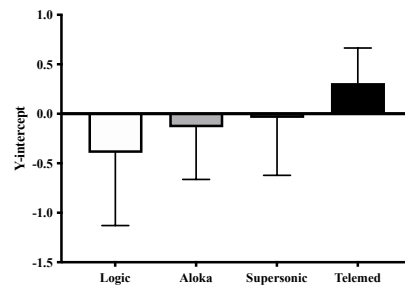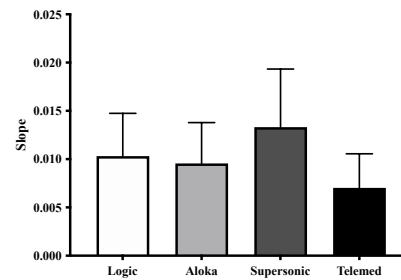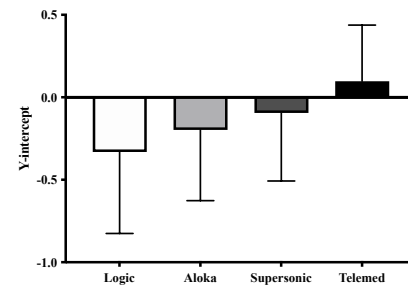

EMCL

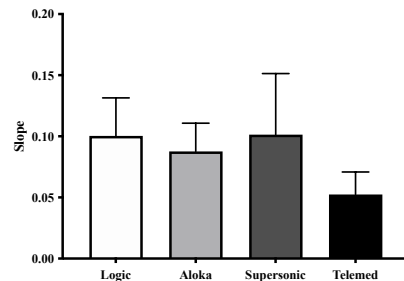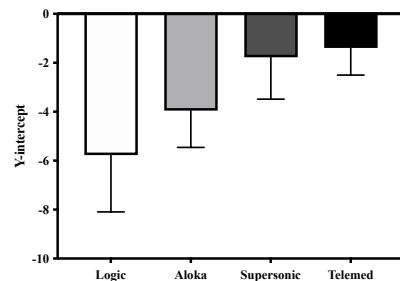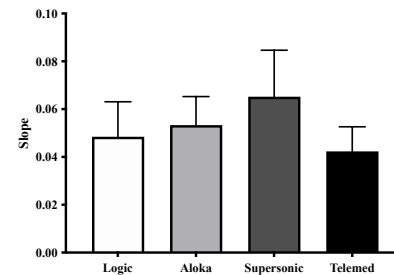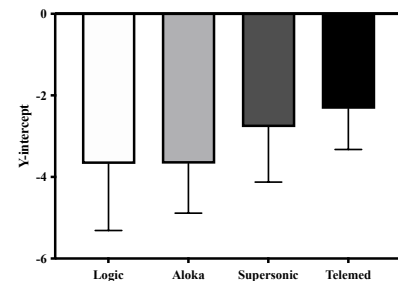

Supplement: Supplementary file 1 [file sensors-23-05282-s001.zip › Supplementary_data/Supplemental data 4.pdf]
